# Vision-Transformer-Based Transfer Learning for Mammogram Classification

**DOI:** 10.3390/diagnostics13020178

**Published:** 2023-01-04

**Authors:** Gelan Ayana, Kokeb Dese, Yisak Dereje, Yonas Kebede, Hika Barki, Dechassa Amdissa, Nahimiya Husen, Fikadu Mulugeta, Bontu Habtamu, Se-Woon Choe

**Affiliations:** 1 Department of Medical IT Convergence Engineering, Kumoh National Institute of Technology, Gumi 39253, Republic of Korea; 2 School of Biomedical Engineering, Jimma University, Jimma 378, Ethiopia; 3 Department of Information Engineering, Marche Polytechnic University, 60121 Ancona, Italy; 4 Biomedical Engineering Unit, Black Lion Hospital, Addis Ababa University, Addis Ababa 1000, Ethiopia; 5 Department of Artificial Intelligence Convergence, Pukyong National University, Busan 48513, Republic of Korea; 6 Department of Basic and Applied Science for Engineering, Sapienza University of Rome, 00161 Roma, Italy; 7 Department of Bioengineering and Robotics, Campus Bio-Medico University of Rome, 00128 Roma, Italy; 8 Center of Biomedical Engineering, Addis Ababa Institute of Technology, Addis Ababa University, Addis Ababa 1000, Ethiopia; 9 Department of IT Convergence Engineering, Kumoh National Institute of Technology, Gumi 39253, Republic of Korea

**Keywords:** transfer learning, transformers, breast cancer, mammography

## Abstract

Breast mass identification is a crucial procedure during mammogram-based early breast cancer diagnosis. However, it is difficult to determine whether a breast lump is benign or cancerous at early stages. Convolutional neural networks (CNNs) have been used to solve this problem and have provided useful advancements. However, CNNs focus only on a certain portion of the mammogram while ignoring the remaining and present computational complexity because of multiple convolutions. Recently, vision transformers have been developed as a technique to overcome such limitations of CNNs, ensuring better or comparable performance in natural image classification. However, the utility of this technique has not been thoroughly investigated in the medical image domain. In this study, we developed a transfer learning technique based on vision transformers to classify breast mass mammograms. The area under the receiver operating curve of the new model was estimated as 1 ± 0, thus outperforming the CNN-based transfer-learning models and vision transformer models trained from scratch. The technique can, hence, be applied in a clinical setting, to improve the early diagnosis of breast cancer.

## 1. Introduction

Breast cancer is the most prevalent cancer in women in the United States, accounting for 30% (or 1 in 3) of all new cases of female cancer each year, except for skin cancers [1,2]. Incidence rates have risen by 0.5% annually in recent years; however, there has been a steady decrease in the number of breast cancer deaths, with an overall decrease of 43% from 1989 to 2020 [1,3]. Better treatment options as well as earlier detection through screening and awareness campaigns are considered the reasons for death rate decline [4,5,6]. Mammography (MG) plays a major role in early detection of breast cancer [7]. MG can detect breast cancer at early stages even with small tumors that cannot be felt as lumps [8]. However, false diagnoses may occur because of the complexity of MGs and the high number of tests performed by radiologists [9]. To provide radiologists with an unbiased perspective, computer-aided detection (CAD), which applies image-processing methods and pattern recognition, has been developed [10]. Studies have demonstrated the value of conventional CAD systems that do not utilize artificial intelligence; however, it remains difficult to accurately detect breast cancer [11]. Nevertheless, the conventional CAD models could not increase the diagnostic efficacy of MG considerably [12,13,14,15]. The significant false-positive rates when employing conventional CAD for anomaly identification in MGs present the main obstacle [16]. False-positive results lead to patient anxiety, unnecessary radiation exposure, pointless biopsies, high callback rates, higher medical expenses, and a greater number of examinations. New and more accurate detection techniques were hence probed, leading to the use of machine learning techniques in the classification of diagnostic images [17,18]. In particular, deep learning (DL) of mammograms is being investigated and applied in large numbers for the early detection of breast cancer in the past few years [19,20,21,22,23]. Convolutional neural network (CNN)-based DL has attracted a lot of attention recently for MGs as it aids in overcoming the constraints of CAD systems (false positives, unnecessary radiation exposure, pointless biopsies, high callback rates, higher medical expenses, and greater number of examinations) [24]. CNNs outperform CAD models in terms of detection accuracy and aid radiologists in making more accurate diagnoses by providing quantitative analyses of complicated lesions [25]. According to previous studies, DL methods significantly lower the likelihood of human error, while diagnosing 85% of the breast cancer cases accurately [26,27,28]. The most recent CNN models are designed to help radiologists discover even the smallest breast tumors in the very early stages, alerting the radiologist to prepare for further interventions [29,30,31].

However, when used on an entire mammogram image, CNNs are computationally expensive due to the multiple convolutions at different feature levels. They focus on a particular area of the image first rather than the entire image and then build up features for the whole image gradually, resulting in expensive computational steps. CNN lacks the ability to handle rotation and scale invariance with no augmentation and fails to encode relative spatial information. To address the issues of failure to encode relative spatial information and the lack of handling rotation and scale invariance, patch-based breast image classifiers are used, where the potential region of interest (ROI) is used rather than the entire image of the breast. This approach has limitations. The first challenge of CNN-based DL models for mammographic breast cancer detection is tumor localization [30]. Most CNN-based DL models use a patch-based approach, whereby a suspected tumor area on a mammogram is cropped and fed into the model [32]. This leads to loss of information from the entire mammogram, resulting in false-positive results [19]. In addition, the patch-based approach is time consuming and computationally expensive [33,34]. The second limitation of the CNN-based approach is that its performance varies based on the size of lesions in an image [35,36]. Thus, the size of the lesion in the region of interest (ROI) affects the performance of CNNs [37]. Third, CNNs require considerable pre-processing to compensate for poor image quality [38]. Owing to reduced visibility, low contrast, poor clarity, and noise, a sizable proportion of abnormalities are misdiagnosed or overlooked [39]. Common pre-processing methods, such as filters, have been suggested to improve image quality, image smoothing, and noise reduction [38]. However, selecting the best method for pre-processing MGs to enhance CNN classification remains a challenge. Fourth, CNNs perform poorly for imbalanced datasets, thus affecting their performance immensely [36,40]. The inequality between positive and negative classes in the training datasets is referred to as dataset imbalance [41]. Directly training CNN models on imbalanced datasets may skew the prediction in favor of classes with a higher number of observations [42]. Finally, CNNs perform poorly in classifying tumors in multi view mammograms, which is a crucial aid in clinical settings [43]. Current CNN models are trained to detect tumors on MGs while ignoring the presence of additional malignancies [44,45,46].

Additionally, finding good datasets for training is a challenge in the medical image domain [47,48], which is true in the case of MG also [19]. This affects the overall success of DL approaches for mammogram classification. Several approaches have been used to compensate for the lack of training image datasets [9,49]. Two widely employed techniques are data augmentation and transfer learning. Data augmentation enables the creation of rearranged image data using the original image, thereby increasing the number and variety of the training image datasets [43]. It includes operations such as noise addition, rotation, translation, contrast, saturation, color augmentation, brightness, scaling, and cropping. Transfer learning utilizes pre-trained weights from selected datasets to be used as a starting point for training on another dataset [19,50]. This approach enables leveraging the knowledge learned from previous tasks for the target task [47]. Almost all CNN-based DL approaches for mammographic breast cancer detection utilize a transfer-learning approach to compensate for the lack of large datasets and to utilize an optimized model with prior feature knowledge for new tasks [51].

In this study, we developed a deep-learning approach for mammographic breast cancer detection using transfer learning based on vision transformers. This study makes two major contributions to the literature. The first is the image-data-balancing module used to solve the class imbalance problem in a mammogram dataset. The dataset utilized for this study is composed of two categories, those from benign and malignant tissues, with unequal sample sizes. In other words, there is a class imbalance that could lead to bias in model learning. To overcome this problem, we propose augmentation-based class balancing. Second, we designed a vision-transformer-based transfer-learning method for mammogram classification. This new transfer-learning approach improves on the shortcomings of CNN-based transfer-learning methods by leveraging the self-attention approach of transformers.

## 2. Related Works

DL based on CNNs is widely employed to aid the early detection of breast cancer using MG. As a result, a few artificial intelligence (AI) tools have been approved by the Food and Drug Administration (FDA) to aid radiologists in decision making. However, owing to the numerous convolution tasks within various network layers, CNNs are computationally complex and require high computational power as the quantity of data increases. Additionally, when analyzing mammograms, CNNs concentrate on a particular region (the region where a tumor is suspected), disregarding the rest of the image, which causes CNNs to miss some crucial details, which would have been discovered if the entire image was examined at once. Vision transformers (ViTs) have recently gained prominence in the field of computer vision, surpassing CNNs in tasks that require natural image classification. Because of their lower computational complexity and ability to overcome the limitations of CNNs in focusing only on a small portion of an image, ViTs outperformed the most advanced CNN models.

The ViT concept is a development of the text-transformer-based original transformer concept. With a minor adjustment in the code to accommodate the various data modalities, it is simply a transformer applied to the image domain. A ViT specifically employs several tokenization and embedding techniques. The general architecture is the same, though. A source image is divided into a collection of image patches known as visual tokens. The visual tokens are incorporated into a collection of fixed-dimension encoded vectors. The transformer encoder network, which is essentially the same as the one in charge of processing the text input, is fed the position of a patch in the image together with the encoded vector. The ViT encoder is composed of several blocks, each of which has three main processing components: the layer norm, the multi-head attention network (MSP), and the multi-layer perceptron (MLP). The model can adjust to differences in the training images thanks to the layer norm, which keeps the training process on track. A network called MSP is in charge of creating attention maps from the provided embedded visual tokens. These attention maps assist the network in concentrating on the image’s most crucial areas, such as the object (s). The MLP is a two-layer classification network with a GELU (Gaussian Error Linear Unit) at the very end. The last MLP block, also referred to as the MLP head, serves as the transformer’s output. SoftMax can be used on this output to provide classification labels (i.e., if the application is image classification).

A few studies have investigated the use of ViTs in classifying mammograms for the early diagnosis of breast cancer. Lee et al. [52] proposed transformer-based DL, which tackles the challenges of mammogram normalization and inter-reader variance in grading. They proposed an approach that uses a photometric transformer network (PTN) as a programmable normalization module to forecast the normalization parameters of input MG. It seamlessly connects to the primary prediction network, allowing for combined learning of the best normalization and density grade. In principle, the PTN resembles a spatial transformer network [53]. However, the PTN seeks to identify a set of photometric transformation parameters that are best for predicting breast density, while the spatial transformer network forecasts suitable geometric transformation parameters. Tulder et al. [45] suggested a novel token-based and pixel-wise cross-view transformer technique and used it on two public MG datasets. The authors suggested an approach based on transformers that join views at the feature map level without requiring pixel-by-pixel correspondences. They used cross-view attention rather than self-attention to transfer information across views, different from how conventional transformers process information inside a single sequence. For image segmentation and breast mass detection in digital mammograms, Su et al. [54] proposed the YOLO–LOGO transformer model. This included two steps: first, they used YoloV5 to detect the breast mass ROI and cropped it directly from the high-resolution image to increase training effectiveness. Thereafter, they used an updated version of the local–global (LOGO) segmentation strategy, which significantly increased the segmentation resolution at the original pixel level. Garrucho et al. [55] evaluated the domain generalization of MG models by comparing the performance of eight cutting-edge detection techniques trained in a single domain, including transformer-based models, and tested them in five unexplored domains. They observed that transformer-based models were more robust and performed better than others in domain generalization of mammograms. Chen et al. [56] used a multi-view transformer (MVT) model to detect breast cancer segments on mammograms. MVT was composed of two main components, the local and global transformer blocks. Local transformer blocks individually analyze data from each view image. In contrast, the global transformer blocks combine data from the four-view mammograms. Self-attention, multi-head attention, and multilayer perceptron were the three main components of the local and global transformer blocks, both of which had the same design.

## 3. Materials and Methods

### 3.1. Dataset

**Figure 1:**
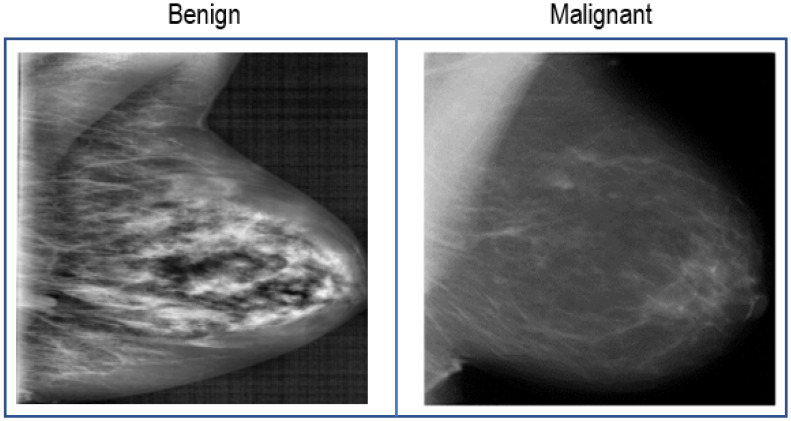
Sample images from the Digital Database for Screening Mammography dataset before augmentation.

In this study, we used the Digital Database for Screening Mammography (DDSM) dataset to train and test our vision-transformer-based transfer-learning system for early identification of breast cancer. This dataset is publicly available and accessible at https://data.mendeley.com/datasets/ywsbh3ndr8/2 [accessed on 12 September 2022]. The dataset includes 13,128 images including 5970 from benign and 7158 from malignant tissues. Sample images from the dataset are shown in Figure 1.

### 3.2. Class Balancing

**Figure 2:**
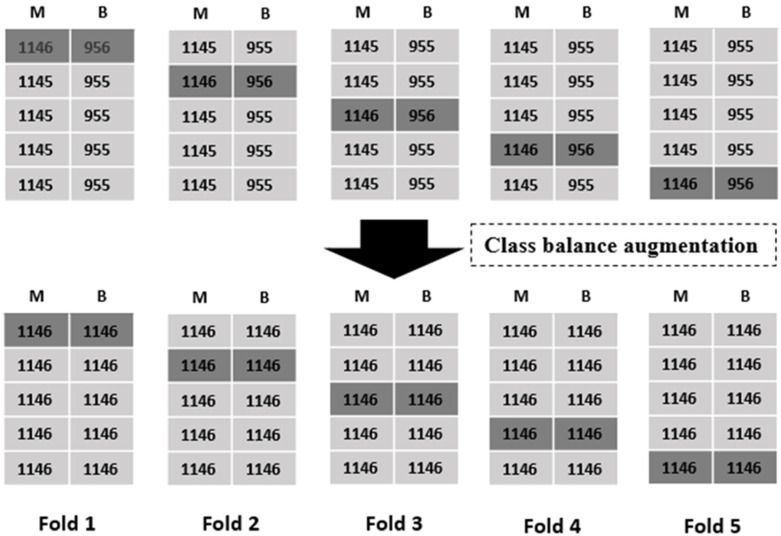
Data class balancing using image augmentation: M, malignant; B, benign.

The dataset retrieved for this study was class imbalanced; that is, the number of images from malignant and benign tissues in the dataset were not equal. The ratio of malignant-to-benign samples in the DDSM dataset was 0.65:0.35. This data distribution may affect the learning of the designed algorithm and had to be fixed first. Thus, we performed a novel data-balancing method using data augmentation. To the best of our knowledge, this data class balancing approach for mammogram images was used for the first time by our group [36]. First, the dataset was categorized into 80% training and 20% testing sets. To balance the dataset for 5-fold cross-validation (nested cross-validation), we used five-image augmentations, including color jitter, gamma correction, horizontal flip, salt-and-pepper, and sharpening, as seen in [36]. The dataset was divided into five folds, each of which included the training and validation datasets. Therefore, in the DDSM dataset, for the first four folds, 1145 images of malignant tumors were present in each fold, whereas 1146 images of malignant tumors were present in the fifth fold. Similarly, for the benign class, the first four folds had 955 images while the fifth fold had 956 images. To balance the data between both classes, we subjected images of the benign class to five image augmentations, whereas malignant mass images underwent only one augmentation. Finally, post-augmentation, 1146 images were present in every fold for both classes of benign and malignant tumors, as shown in Figure 2.

### 3.3. Preprocessing

The pixel sizes of the mammograms in the dataset varied considerably; therefore, we resized all images to a size of 224 × 224 pixels, which is the preferred size for patch generation from the input images.

### 3.4. The Proposed Method

In this study, we performed a vision-transformer-based transfer-learning method to classify mammograms as being from benign or malignant tissues. Therefore, vision transformer models pre-trained on natural images (ImageNet dataset) were used for mammogram classification.

#### 3.4.1. Vision Transformer Architecture

Vision transformers are derived from the original transformer model used in the natural language processing (NLP) model, where the input is a one-dimensional sequence of word tokens. However, images are two-dimensional, and vision transformer models partition images into smaller two-dimensional patches and input the patches as word tokens, as performed by the original NLP transformer models. The input image of height H, width W, and number of channels C, is divided into smaller two-dimensional patches to arrange the input image data in a way similar to how the input is structured in the NLP domain. This produces $N=\frac{HW}{P^{2}}$ patches with a pixel size of P×P [57]. Prior to providing the patches to the transformer encoder, flattening, sequence imbedding, learnable embedding, and patch embedding were performed in the following order:

- Every patch was flattened into a vector, $X_{p}^{n}$, with a length of $P^{2}\times C$, for n=1,…N.
- Mapping the flattened patches to D dimensions using a trainable linear projection, E, produced a series of embedded image patches.
- The sequence of the embedded image patches was prefixed with a learnable class embedding $X_{class}$. The $X_{class}$ values correspond to the classification outcome Y.
- Finally, one-dimensional positional embeddings $E_{pos}$, which are also learned during training, are added to the patch embeddings to add positioning information to the input.

The embedding vectors produced as a result of the aforementioned operations are given by $z_{o}$ (1):

(1) $$z_{o}=\left[X_{class};\;X_{p}^{1}E;\ldots ;X_{p}^{N}E\right]+E_{pos}$$

We fed $z_{o}$ to the transformer–encoder network, which is a stack of L identical layers, to conduct the classification. The classification head was then fed with the value of $X_{class}$ at the $L^{th}$ layer of the encoder output. A MLP with a single hidden layer was used to implement the classification head during pretraining, and a single linear layer was used during fine tuning. The MLP implements the GELU nonlinearity, serving as the classification head.

**Figure 3:**
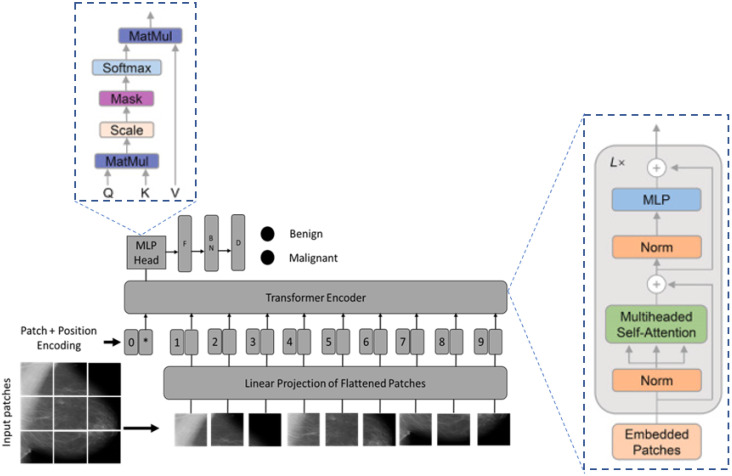
The vision transformer-based transfer learning architecture for mammogram breast image detection: MLP, multilayer perceptron; F, flattening; BN, batch normalization; D, dense; L, linear; Q, query; K, key; V, value; *, extra learnable (class) embedding.

Overall, the vision transformer used the encoder components of the original NLP transformer architecture. The encoder receives a sequence of embedded picture patches of size 16 × 16 as input, together with positional data, and a learnable class embedding suspended to the sequence. The smaller the size of the patch, the higher the performance will be and the higher the computational cost will be. Thus, 16 × 16 patch size was chosen as in [58] because of its robustness against performance degradation and computational complexity. The learnable class-embedding value is sent to a classification head coupled to the output of the encoder, which uses it to produce a classification output depending on its state. Figure 3 shows the general structure of the vision-transformer-based transfer-learning architecture. The original vision transformer model, pre-trained on the ImageNet dataset, was used in such a way that the last layer was replaced with a flattening layer followed by batch normalization and an output dense layer.

#### 3.4.2. Transfer Learning

Transfer learning was employed such that vision transformer models pre-trained on the large ImageNet natural image dataset were utilized as a starting point to train the mammogram dataset. The objective was to use the vision transformer’s knowledge from the large natural image dataset to classify breast mammograms into two classes: those from benign and malignant tissues. For this, we detached the pre-trained prediction head and replaced it with a D×K feedforward layer, where K=2 is the total number of classes in the downstream direction. Here, with transfer learning, we sought to enhance the learning of the target function $f_{t}\left(\cdot \right)$ in the target domain $D_{t}$, utilizing the knowledge from the source domain $D_{s}$, and the learning task, $T_{s}$. The ImageNet dataset has m training samples $\left\{\left(x^{1},\;y^{1}\right),\ldots ,\left(x^{i},\;y^{i}\right),\ldots ,\left(x^{m},\;y^{m}\right)\right\}$, where $x^{i}$ and $y^{i}$ represent the $i^{th}$ input and label, respectively. Thereafter, the weights of the ImageNet pre-trained vision transformer model $W_{0}$, were utilized as a starting point during transfer learning to generate $W_{1}$ by minimizing the objective function in (2), where $\langle y^{ij}|x^{ij},\;W_{0},W_{1},b\rangle$ is the Softmax output probability function, and b is the bias.

(2) $$J\left(\langle W_{1},\;b|W_{0}\rangle \right)=\frac{-1}{mn}\sum_{i=1}^{m}\sum_{j=1}^{m}y^{ij}\log\left(P\langle y^{ij}|x^{ij},\;W_{0},W_{1},b\rangle \right)$$

**Figure 4:**
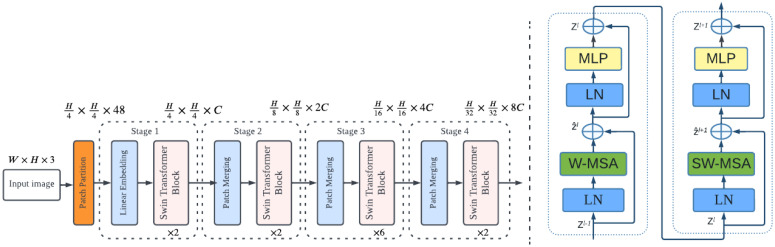
Swin transformer architecture: *H*, height; *W*, width; *C*, channel; LN, layer normalization; MLP, multilayer perceptron; *Z*, output features; W-MSA, window based multi-head self-attention; SW-MSA, shifted window based multi-head self-attention.

In this study, we utilized three state-of-the-art Vision Transformer models: the vision transformer (ViT) model proposed by Dosovitskiy et al. [58], the Swin transformer (Swin-T) model proposed by Liu et al. [59], and the pyramid vision transformer (PVT) model proposed by Wang et al. [60]. Swin-T improves locality using local or window attention by applying self-attention to nonoverlapping windows. By gradually integrating the windows, window-to-window communication in the following layer creates a hierarchical representation, as shown in Figure 4. There are four variants of Swin transformer, Swin-tiny, Swin-small, Swin-base, and Swin-large, but in our case, we utilized Swin-small and Swin-base owing to improved performance and reduced computational complexity.

**Figure 5:**
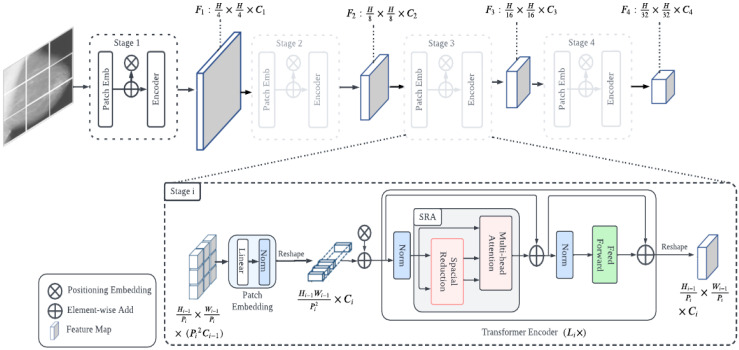
Pyramid vision transformer architecture: *H*, height; *W*, width; *C*, channel; *P_i_*, *i*-th stage patch size; *F*, feature map; *L_i_*, transformer–encoder layer; SRA, spatial-reduction attention.

PVT uses a type of self-attention known as spatial-reduction attention (SRA), which is characterized by a spatial reduction in both keys and values, to obtain the quadratic complexity of the attention mechanism [60]. SRA gradually reduced the spatial dimensionality of the characteristics across the entire model. In addition, it applied positional embeddings to all transformer blocks, strengthening the idea of order. The PVT architecture is shown in Figure 5. There are four variants of PVT, PVT-tiny, PVT-small, PVT-medium, and PVT-large, but in our case, we utilized PVT-medium and PVT-large for improved performance.

### 3.5. Experimental Settings

The performance of the proposed method was evaluated using five experimental settings. The first is a comparison of the performance of the proposed transfer-learning method using three state-of-the-art vision transformer architectures. Second, we trained vision transformer models on the mammogram dataset from scratch using these three architectures and compared them with their transfer learning counterparts. Third, we compared transfer learning using vision transformers with CNNs. In the fourth experimental setting, the computational cost of each vision transformer model was evaluated. Fifth, we compared the performance of the proposed method with those using previous methods on the same dataset.

### 3.6. Implementation Details

The models in this study were trained for 50 epochs with a learning rate of 0.0001, using the Adam optimizer. These parameters were chosen based on prior studies on the same dataset and hardware and software settings [19]. We applied an exponential decay and a batch size of 64. We divided our datasets into training and testing groups in an 8:2 ratio. For the vision transformer models, GELU was used as an activation function, together with an L2 regularizer. A rectified linear unit (ReLu) was used in the CNNs, along with an L2 regularizer. To prevent bias in the results, the same parameter settings were used for all comparisons. Five-fold cross-validation was used to compare the model performances. RTX 3090 GPUs were used to implement the proposed transfer learning model. We used Python programming language version 3.6 on TensorFlow framework.

### 3.7. Performance Metrics

Model performances were determined in terms of machine learning quantitative performance metrics and statistical measures. The metrics included accuracy, area under the receiver operating curve (AUC), F1-score, precision, recall, Matthew’s correlation coefficient (MCC) [61], and kappa scores, all of which were calculated with a 95% confidence interval. Table 1 provides the details of the performance metrics.

## 4. Results

The proposed vision-transformer-based transfer-learning model exhibited superior performance on the DDSM dataset, as shown in Table 2. Six of the models used were from three different vision-transformer-based architectures and performed uniformly in terms of all the metrics used to evaluate performance. Consequently, the proposed vision-transformer-based transfer-learning model provided an accuracy, AUC, F1 score, precision, recall, MCC, and kappa value of 1 ± 0 on the DDSM dataset. This provides strong evidence that vision-transformer-based transfer learning is effective in improving the DL approach for breast mammograms, thereby improving the early diagnostic techniques for breast cancer.

**Figure 6:**
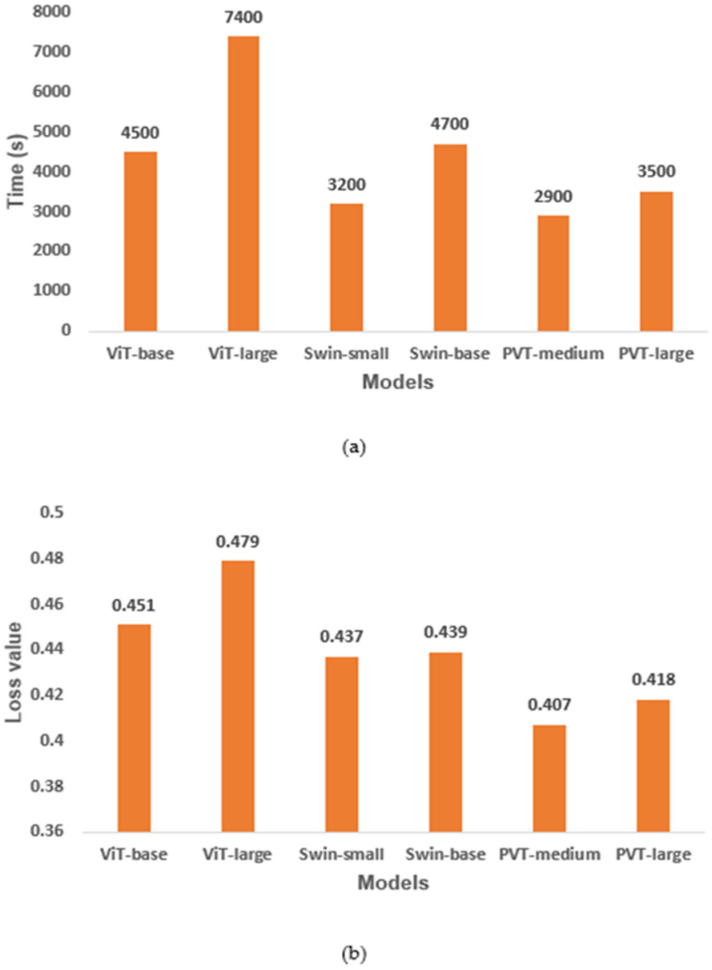
Training time and loss value of the vision-transformer-based transfer-learning models: (**a**) training time in seconds (s), and (**b**) loss values. ViT, vision transformer; PVT, pyramid vision transformer.

Figure 6 depicts the training time taken in seconds (s) (Figure 6a) and loss value (Figure 6b) of each model. Even though the loss value for the six models is between 0.4 and 0.5, the training time needed for each model varies. The ViT-large model needed 7400 s, being the slowest for training. On the other hand, the PVT-medium model took only 2900 s, being the fastest model to train on the DDSM dataset. This shows that some models take longer time to train to achieve the best performance, as in the case of ViT-large, while others need less training time to achieve the same performance, as in the case of PVT-medium. This is critical while choosing a model to deploy, especially in clinical settings where large number of images are processed every day.

**Figure 7:**
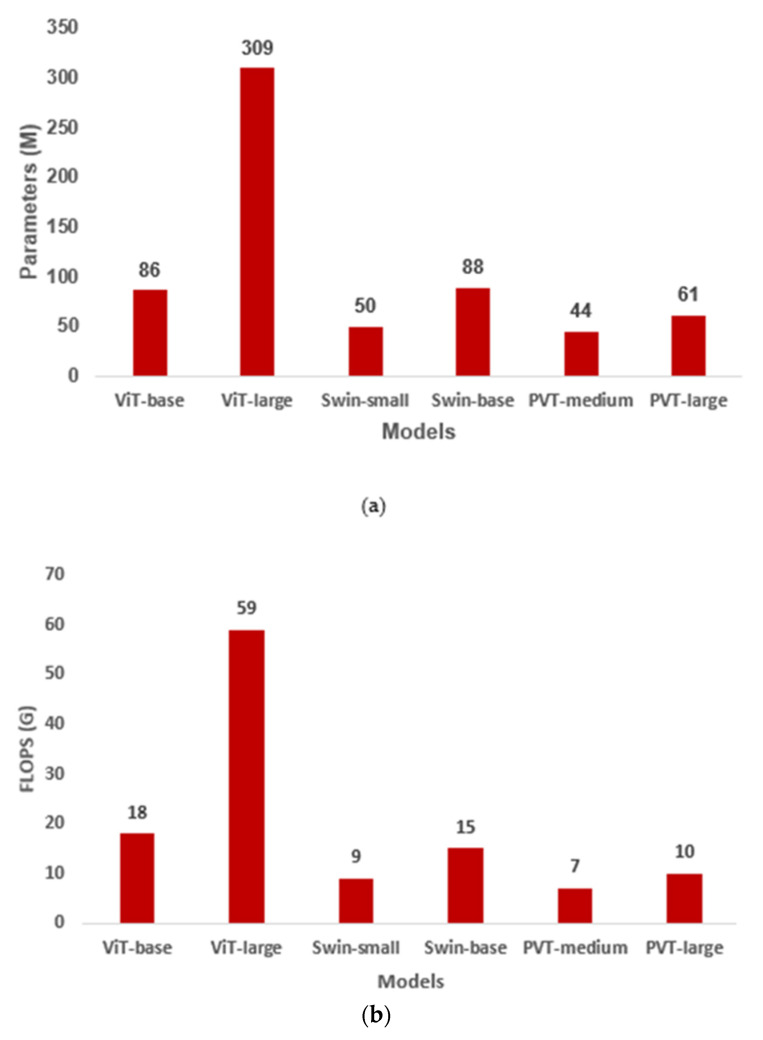
Computational cost performance comparison of different transfer learning models on DDSM data: (**a**) number of parameters in millions (M) and (**b**) FLOPS value in giga (G). ViT, vision transformer; PVT, pyramid vision transformer.

An investigation was conducted to determine the computational complexity of the proposed method. To do so, we used floating point operations per second (FLOPS) to compare the computational costs of the different vision-transformer-based transfer-learning models. FLOPS is a measure of the number of operations needed to run a single instance of a certain model. For instance, how many operations are required to train a single instance of ViT model. The larger the FLOPS, the higher the computational cost; the lower the FLOPS, the lower the computational cost. Thus, a model with a smaller FLOPS is preferred. Figure 7 depicts the number of parameters in millions (M) (Figure 7a) trained for each model and their corresponding FLOPS in gigas (G) (Figure 7b) for all six vision-transformer-based transfer-learning models. As can be seen from the figure, models with a large number of parameters have a larger FLOPS, and vice versa. In our case, the PVT-medium with 44 million parameters had the smallest FLOPS of 7G. In contrast, ViT-large with 309 million parameters had the highest FLOPS of 59G. Therefore, the PVT-medium with the smallest value of FLOPS was effective for vision-transformer-based transfer learning on the DDSM dataset, although its performance in terms of accuracy is the same as that of the other five models.

We trained a vision transformer model built from scratch, as in Dosovitskiy et al. [58], Liu et al. [59], and Wang et al. [53] to compare their performance against pre-trained vision transformer models (the proposed transfer learning method) for classifying breast images. We used pre-trained vision transformer models, and the final layer was changed to reflect the number of classes we wanted to categorize into (two in our case). Apart from that, we used the original models (which have the same number of layers as the original models utilized in this paper) as they are. For a fair comparison, we utilized the same optimizers and their corresponding learning rates in other models trained from scratch, as those in the proposed model. Table 3 shows the results of the transformer models on the DDSM dataset trained from scratch. PVT-medium model provided the highest performance result among all the vision transformer models trained from scratch. It exhibited an accuracy, AUC, F1 score, precision, recall, MCC, and kappa score of 0.78 ± 0.02, 0.77 ± 0.02, 0.78 ± 0.01, 0.78 ± 0.02, 0.78 ± 0.02, 0.77 ± 0.01, and 0.77 ± 0.02, respectively. This result is far inferior to the results achieved by the vision-transformer-based transfer-learning models depicted in Table 2. Hence, vision-transformer-based transfer-learning models provide improved performance compared with vision transformer models trained from scratch on the DDSM dataset.

To compare the performance of the proposed vision-transformer-based transfer learning with CNN-based transfer learning, we ran experiments using state-of-the-art CNN models, with the same setting as in vision transformer models, except for using ReLu and GELU as the activation functions for CNN-based models and vision-transformer-based models, respectively. The results of the performance of the CNN-based transfer learning models on the DDSM dataset are presented in Table 4. Compared with the results achieved by the vision-transformer-based transfer-learning models in Table 2, which has the highest AUC of 1 ± 0, the results of the CNN-based transfer-learning models, with the highest AUC of 0.95 ± 0.01 for ResNet50, indicate that CNN-based transfer learning models perform much poorer. This indicates that vision transformers are better for mammograms than CNNs are.

## 5. Discussion

Vision-transformer-based transfer learning for breast mammogram classification was developed in this study. We implemented image-augmentation-based class-wise data balancing to compensate for the imbalance in the number of benign and malignant samples within the DDSM dataset. This helps the proposed model to avoid bias to a given class with a greater amount of data and negatively affects the detection outcome. The state-of-the-art vision transformer architectures, including the vision transformer model proposed by Dosovitskiy et al. [58], the Swin vision transformer model proposed by Liu et al. [59], and the pyramid vision transformer model proposed by Wang et al. [60], were utilized to evaluate the performance of the developed vision-transformer-based transfer learning for breast mammogram classification. Consequently, the vision-transformer-based transfer-learning approach provided the highest quantitative and statistical measures for classifying breast mammograms as being from benign or malignant tissues. This proves the effectiveness and quality of the vision-transformer-based transfer-learning approach for detecting breast cancer from mammograms. The prime reason for the better performance of vision transformers is the ability to capture global information from the early layers and the deep self-attention mechanism that enables features in each patch to be carefully analyzed for decision making. Additionally, our study showed that vision transformer models are more effective when used for transfer learning on the DDSM dataset than training the models from scratch, because of the small number of images in the DDSM dataset. DL models require a large amount of data for training and a large number of parameters to be trained, which results in the overfitting of the models in the case of a small training dataset, such as the DDSM data. Therefore, transfer learning provided better results as it used weights that were pre-trained on large datasets, such as the ImageNet dataset, and leverages that knowledge to learn from small datasets, such as DDSM, during training. We further investigated the effectiveness of vision-transformer-based transfer learning by comparing it directly with CNN-based transfer learning for classifying breast mammograms as being from benign or malignant tissues. To summarize, we observed that vision-transformer-based transfer learning outperformed CNN-based transfer learning of the DDSM dataset. Moreover, PVT-based transfer-learning models were computationally less expensive, providing the same performance as those of other models, including ViTs with a lower computational cost for breast mammogram classification. Finally, we compared our approach with models from published works and found that our approach produced the best performance results (Table 5). Details about the models in Table 5 can be found in Section 2.

## 6. Conclusions

We have presented a vision-transformer-based transfer-learning approach for breast mammogram classification. A detailed evaluation using different vision transformer models and variants has been performed. Consequently, we found that vision-transformer-based transfer learning is effective for breast mammogram image classification, providing superior performance with less computational complexity. Vision transformer-based transfer learning outperformed convolutional neural network-based transfer learning for breast mammogram classification. However, this result was obtained from training on a single dataset obtained from one source, and further studies utilizing different datasets from different sources should be considered to generalize the result obtained in this study. Future studies should also consider the use of various deep learning parameters to investigate their effect on vision-transformer-based transfer learning for breast mammogram image classification.

## Figures and Tables

**Table 1:** Performance metrics: TP, true positive; TN, true negative; FP, false positive; FN, false negative.

Metrics	Formula
Accuracy	$\frac{TP+TN}{TP+FP+FN+TN}$
Precision	$\frac{TP}{\left(TP+FP\right)}$
Recall	$\frac{TP}{\left(TP+FN\right)}$
F1 score	$\frac{TP}{TP+\frac{1}{2}\left(FP+FN\right)}$
MCC score	$\frac{TN\times TP-FN\times FP}{\surd \left(TP+FP\right)\left(TP+FN\right)\left(TN+FP\right)\left(TN+FN\right)}$
Kappa score	$\frac{2\times \left(TP\times TN-FN\times FP\right)}{\left(TP+FP\right)\times \left(FP+TN\right)+\left(TP+FN\right)\times \left(FN+TN\right)}$

**Table 2:** Results of vision-transformer-based transfer learning for breast cancer detection from mammograms: AUC, area under receiver operating characteristic curve; MCC, Matthew’s correlation coefficient.

Architecture	Model	Accuracy (95%)	AUC (95%)	F1 Score (95%)	Precision (95%)	Recall (95%)	MCC (95%)	Kappa (95%)
Vision transformer	ViT-base	1 ± 0	1 ± 0	1 ± 0	1 ± 0	1 ± 0	1 ± 0	1 ± 0
	ViT-large	1 ± 0	1 ± 0	1 ± 0	1 ± 0	1 ± 0	1 ± 0	1 ± 0
Swin transformer	Swin-small	1 ± 0	1 ± 0	1 ± 0	1 ± 0	1 ± 0	1 ± 0	1 ± 0
	Swin-base	1 ± 0	1 ± 0	1 ± 0	1 ± 0	1 ± 0	1 ± 0	1 ± 0
Pyramid vision transformer	PVT-medium	1 ± 0	1 ± 0	1 ± 0	1 ± 0	1 ± 0	1 ± 0	1 ± 0
	PVT-large	1 ± 0	1 ± 0	1 ± 0	1 ± 0	1 ± 0	1 ± 0	1 ± 0

**Table 3:** Results of vision transformer methods trained from scratch for breast cancer detection from mammograms: AUC, area under receiver operating characteristic curve; MCC, Matthew’s correlation coefficient.

Architecture	Model	Accuracy (95%)	AUC (95%)	F1 Score (95%)	Precision (95%)	Recall (95%)	MCC (95%)	Kappa (95%)
Vision transformer	ViT-base	0.74 ± 0.02	0.73 ± 0.03	0.74 ± 0.01	0.74 ± 0.01	0.74 ± 0.01	0.73 ± 0.03	0.73 ± 0.02
	ViT-large	0.72 ± 0.04	0.72 ± 0.02	0.72 ± 0.03	0.72 ± 0.04	0.72 ± 0.03	0.71 ± 0.02	0.72 ± 0.01
Swin transformer	Swin-small	0.75 ± 0.02	0.75 ± 0.03	0.75 ± 0.01	0.75 ± 0.02	0.75 ± 0.02	0.74 ± 0.03	0.74 ± 0.02
	Swin-base	0.76 ± 0.01	0.75 ± 0.02	0.75 ± 0.02	0.75 ± 0.01	0.76 ± 0.01	0.75 ± 0.01	0.75 ± 0.02
Pyramid vision transformer	PVT-medium	0.78 ± 0.02	0.77 ± 0.02	0.78 ± 0.01	0.78 ± 0.02	0.78 ± 0.02	0.77 ± 0.01	0.77 ± 0.02
	PVT-large	0.77 ± 0.03	0.77 ± 0.01	0.77 ± 0.02	0.77 ± 0.02	0.77 ± 0.02	0.77 ± 0.01	0.77 ± 0.01

**Table 4:** Results of CNN-based transfer learning for breast cancer detection from mammograms: AUC, area under receiver operating characteristic curve; MCC, Matthew’s correlation coefficient.

Architecture	Model	Accuracy (95%)	AUC (95%)	F1 Score (95%)	Precision (95%)	Recall (95%)	MCC (95%)	Kappa (95%)
ResNet	ResNet50	0.95 ± 0.01	0.96 ± 0.01	0.95 ± 0.01	0.95 ± 0.01	0.95 ± 0.01	0.94 ± 0.01	0.94 ± 0.02
	ResNet101	0.95 ± 0.01	0.95 ± 0.02	0.95 ± 0.01	0.95 ± 0.01	0.95 ± 0.01	0.94 ± 0.02	0.94 ± 0.02
EfficientNet	EfficientNetB0	0.94 ± 0.02	0.94 ± 0.01	0.94 ± 0.01	0.94 ± 0.01	0.94 ± 0.01	0.93 ± 0.03	0.93 ± 0.02
	EfficientNetB2	0.95 ± 0.01	0.95 ± 0.01	0.95 ± 0.01	0.95 ± 0.01	0.95 ± 0.01	0.95 ± 0.01	0.95 ± 0.01
InceptionNet	InceptionNetV2	0.93 ± 0.02	0.93 ± 0.01	0.93 ± 0.02	0.93 ± 0.02	0.93 ± 0.02	0.93 ± 0.03	0.92 ± 0.02
	InceptionNetV3	0.94 ± 0.01	0.94 ± 0.01	0.94 ± 0.01	0.94 ± 0.01	0.94 ± 0.01	0.93 ± 0.02	0.93 ± 0.02

**Table 5:** Performance of published works on mammogram breast cancer detection using transformers: DDSM, Digital Database for Screening Mammography; AUC, area under receiver operating characteristic curve.

Paper	Purpose	Dataset	AUC
Lee et al. [52]	Classification	Private	0.9663 ± 0.033
Tulder et al. [45]	Classification	DDSM	0.803 ± 0.003
Su et al. [54]	Detection	DDSM	0.65
Garrucho et al. [55]	Detection	OPTIMAM	0.948
Chen et al. [56]	Classification	Private	0.818 ± 0.039
Current work	Classification	DDSM	1 ± 0

## Data Availability

In this study, we used the publicly available breast mammogram dataset from the Digital Database for Screening Mammography (DDSM) (available at https://data.mendeley.com/datasets/ywsbh3ndr8/2 [accessed on 12 September 2022]).
